# Hepatic steatosis and liver fat contents in liver transplant recipients are associated with serum adipokines and insulin resistance

**DOI:** 10.1038/s41598-020-69571-1

**Published:** 2020-07-29

**Authors:** Ahad Eshraghian, Saman Nikeghbalian, Alireza Shamsaeefar, Kourosh Kazemi, Mohammad Reza Fattahi, Seyed Ali Malek-Hosseini

**Affiliations:** 1 Shiraz Transplant Center, Abu-Ali Sina Hospital, Shiraz, Iran; 2 Shiraz Transplant Center, Abu-Ali Sina Hospital, Shiraz University of Medical Sciences, Shiraz, Iran; 3 Department of Hepatobiliary Pancreatic and Transplant Surgery, Shiraz University of Medical Sciences, Shiraz, Iran; 4 Department of Gastroenterology and Hepatology, Shiraz University of Medical Sciences, Shiraz, Iran

**Keywords:** Liver, Non-alcoholic fatty liver disease

## Abstract

Our data about pathogenesis of hepatic steatosis after liver transplantation is scarce. This study aimed to investigate the association between serum adipokines and insulin resistance with hepatic steatosis in liver transplant recipients. We investigated the association between insulin resistance, serum adiponectin, insulin, and leptin with hepatic steatosis in a cohort of liver transplant recipients. Homeostatic model assessment of insulin resistance 2 (HOMA 2-IR) was used for estimation of insulin resistance. Hepatic steatosis was determined using ultrasound and controlled attenuation parameter (CAP). A total of 178 patients were included. 79 patients (44.4%) had hepatic steatosis. Serum adiponectin (OR: 0.912; 95% CI 0.869–0.957; P < 0.001), serum leptin (OR: 1.060; 95% CI 1.017–1.102; P = 0.005), HOMA2-IR (OR: 1.671; 95% CI 1.049–2.662; P = 0.031), and post-transplant diabetes mellitus (PTDM) (OR: 5.988; 95% CI 1.680–21.276; P = 0.006) were independently associated with hepatic steatosis after liver transplantation. CAP values were negatively correlated with serum adiponectin (P = 0.011) and positively correlated with serum insulin (P = 0.001), leptin (P < 0.001) and HOMA2-IR (P < 0.001). Insulin resistance and alterations in adipokines might have central role in pathogenesis of hepatic steatosis after liver transplantation and can be targeted for diagnostic and therapeutic purposes.

## Introduction

Non-alcoholic fatty liver disease (NAFLD) is rapidly surpassing other indications of liver transplantation during recent years and is going to become the leading cause of liver transplantation worldwide^1^. This is mainly due to the increasing prevalence of NAFLD secondary to the epidemics of metabolic syndrome and obesity^2^. NAFLD is not only an increasing cause of liver transplantation but also might occur after transplantation as recurrence of the disease or de novo hepatic steatosis^3^. The prevalence of hepatic steatosis after liver transplantation has been ranged from 30–60% in different studies^4,5^.

While pathogenesis of NAFLD and nonalcoholic steatohepatitis (NASH) has been thoroughly investigated in recent years^6,7^, our data regarding pathogenesis of post-transplant hepatic steatosis is limited. Several risk factors for hepatic steatosis after liver transplantation have been hypothesized. Post-transplant metabolic syndrome and obesity is prevalent after liver transplantation promoting the process of hepatic steatosis^8^. Weight gain is occurred in the majority of patients after liver transplantation irrespective of the underlying cause of cirrhosis and is probably one of the main contributing factors for hepatic steatosis after liver transplantation^9,10^. Hyperlipidemia, post-transplant diabetes mellitus (PTDM) and hypertension are traditional risk factors for fatty liver disease that might occur after liver transplantation^11,12^. Insulin resistance has a central role in the pathogenesis of NAFLD. Insulin resistance is involved in progression of NAFLD by increasing release of free fatty acids from the adipose tissue and de novo lipogenesis within hepatocytes secondary to hyperinsulinemia^13,14^. Insulin resistance can also cause flux of free fatty acids from adipocytes in to the liver^14^. Serum adipokines including adiponectin and leptin have been reported to be involved in the pathogenesis of NAFLD. Serum leptin level is increased and serum adiponectin is decreased in patients with NAFLD in parallel to the severity of disease^15^.

To date, only few data are available about association of post-transplant hepatic steatosis with serum adipokines and insulin resistance as the main mechanisms of hepatic steatosis. In this study we aimed to investigate the impact of insulin resistance and serum adipokines on post-transplant hepatic steatosis.

## Results

### Association of hepatic steatosis with serum adipokines and insulin resistance after liver transplantation

**Figure 1:**
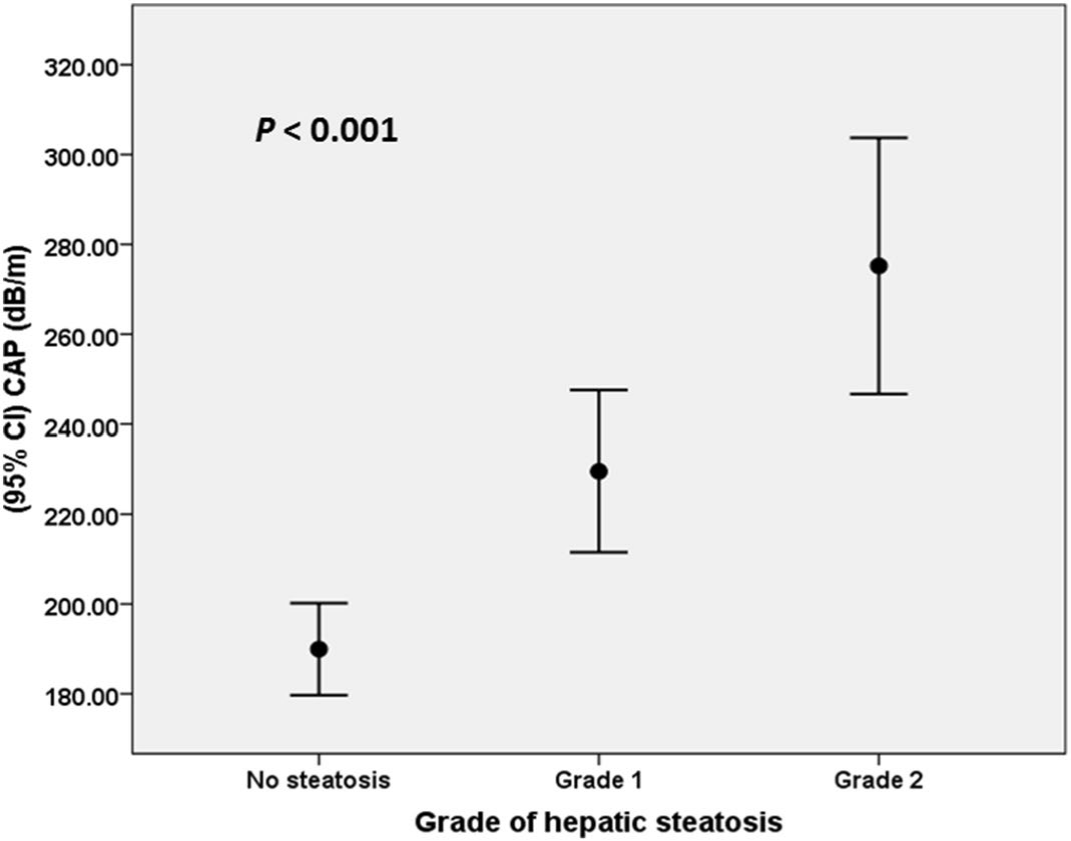
Correlation of controlled attenuation parameter (CAP) values with steatosis grade in ultrasound.

178 liver transplant recipients were included in the study. There were 99 men (55.6%) and 79 women (44.4%). Baseline characteristics of patients are outlined in Table 1. The meantime for evaluation of fatty change after liver transplantation and blood sampling was 38.83 ± 34.43 months. Using ultrasound, 79 patients (44.4%) were diagnosed to have hepatic steatosis after liver transplantation. Twenty eight patients (35%) had grade 1 steatosis, 29 patients (36.7%) had grade 2 steatosis and 22 patients were diagnosed to have grade 3 steatosis in ultrasound. CAP values were statistically different between patients without steatosis (189.95 ± 51.88 dB/m), with grade 1 stetaosis (229.52 ± 65.40 dB/m), and with grade 2 stetaosis (275.20 ± 39.87 dB/m) (P < 0.001) in ultrasound (Fig. 1). Univariate analysis of risk factors for hepatic steatosis after liver transplantation are outlined in Table 2. Mean serum concentration of adiponectin after liver transplantation was 13.30 ± 10.59 µg/ml in patients with steatosis compared to 24.91 ± 17.42 µg/ml in patients without steatosis (P < 0.001). Serum concentration of leptin (20.64 ± 22.02 ng/ml versus 9.97 ± 11.97 ng/ml) and insulin (15.81 ± 17.73 µU/ml versus 8.80 ± 7.07 µU/ml) were significantly higher in patients with hepatic steatosis compared to those without hepatic steatosis (P < 0.001). In regression analysis, serum adiponectin (OR: 0.912; 95% CI 0.869-0.957; P < 0.001), serum leptin (OR: 1.060; 95% CI 1.017–1.102; P = 0.005), HOMA2-IR as a marker of insulin resistance (OR: 1.671; 95% CI 1.049–2.662; P = 0.031), and PTDM (OR: 5.988; 95% CI 1.680–21.276; P = 0.006), were independently associated with hepatic steatosis after liver transplantation (Table 2).

**Table 1:** Clinical characteristics of the study patients.

Variables	Patient characteristic
Age (years)	44.51 ± 12.60
Men/women	99/79 (55.6%–44.4%)
Weight (kg)	69.99 ± 14.66
Height (cm)	166.63 ± 11.81
WC (cm)	94.37 ± 12.22
HC (cm)	102.74 ± 10.77
BMI (kg/m^2^)	25.60 ± 9.24
Underlying liver disease, n (%)	
HBV	33
Cryptogenic	17
AIH	24
PSC	35
NASH	33
HCV	5
Others	31
PTDM, n (%)	65 (36.5)
HLP, n (%)	51 (28.7)
HTN, n (%)	41 (23)

*BMI* Body mass index, *WC* waist circumference, *HC* hip circumference, *HBV* hepatitis B virus, *HCV* hepatitis C virus, *AIH* autoimmune hepatitis, *PSC* Primary sclerosing cholangitis, *NASH* non-alcoholic steatohepatitis, *PTDM* Post transplant diabetes mellitus, *HLP* Hyperlipidemia, *HTN* hypertension.

**Table 2:** Univariate and multivariate logistic regression analysis of serum adipokines and other risk factors of hepatic steatosis after liver transplantation.

	Univariate analysis			Multivariate analysis		
	With steatosis	Without steatosis	P-value	Odds ratio	95% CI	P-Value
Age (year)	47.30 ± 11.76	42.36 ± 12.90	0.009	1.020	0.935–1.027	0.402
Men/women, n (%)	50 (63)/29 (37)	49 (49)/50 (51)	0.057			
BMI (Kg/m^2^)	27.58 ± 5.02	24.08 ± 11.38	0.014	1.006	0.932–1.088	0.883
WC (cm)	101.50 ± 11.92	88.67 ± 9.15	< 0.001	1.013	0.896–1.141	0.856
HC (cm)	108.88 ± 11.07	97.84 ± 7.61	< 0.001	1.058	0.936–1.196	0.369
Triglyceride (mg/dL)	178.40 ± 87.36	124.20 ± 66.28	< 0.001	1.005	0.996–1.014	0.278
HDL (mg/dL)	42.64 ± 12.66	48.34 ± 16.51	0.021	1.003	0.956–1.039	0.997
Cholesterol (mg/dL)	181.41 ± 55.96	166.03 ± 53.18	0.085			
LDL (mg/dL)	97.55 ± 36.38	90.63 ± 35.61	0.076			
FBS (mg/dL)	129.77 ± 52.19	104.14 ± 36.42	< 0.001			
AST (IU/L)	23.92 ± 11.88	32.51 ± 34.38	0.059			
ALT (IU/L)	27.22 ± 13.01	39.30 ± 50.98	0.068			
Time to study (months)	42.21 ± 36.05	35.76 ± 32.98	0.216			
PTDM, n (%)	41 (53.2)	24 (25)	< 0.001	5.988	1.680–21.276	0.006
HTN, n (%)	24 (32.4)	17 ( 18.3)	0.035	1.729	0.548–5.458	0.381
HLP, n (%)	33 (43.4)	18 (18.8)	< 0.001	0.430	0.102–1.808	0.250
NASH, n (%)	24 (30.4)	9 (9.4%)	< 0.001	0.802	0.171–3.747	0.779
Adiponectin (µg/ml)	13.30 ± 10.59	24.91 ± 17.42	< 0.001	0.912	0.869–0.957	< 0.001
Leptin (ng/ml)	20.64 ± 22.02	9.97 ± 11.97	< 0.001	1.060	1.017–1.102	0.005
Insulin (µU/ml)	15.82 ± 17.73	8.80 ± 7.07	< 0.001			
HOMA2-IR	2.04 ± 1.80	1.20 ± 0.87	< 0.001	1.671	1.049–2.662	0.031

*BMI* Body mass index, *WC* waist circumference, *HC* Hip circumference, *PTDM* post transplant diabetes mellitus, *HLP* Hyperlipidemia, *HTN* Hypertension, *HOMA-IR* Homeostatic model for insulin resistance, *FBS* Fasting blood sugar, *AST* Aspartate aminotransferase, *ALT* Alanine aminotransferase, *NASH* non-alcoholic steatohepatitis, *HDL* High density lipoprotein, *LDL* Low density lipoprotein.

**Figure 2:**
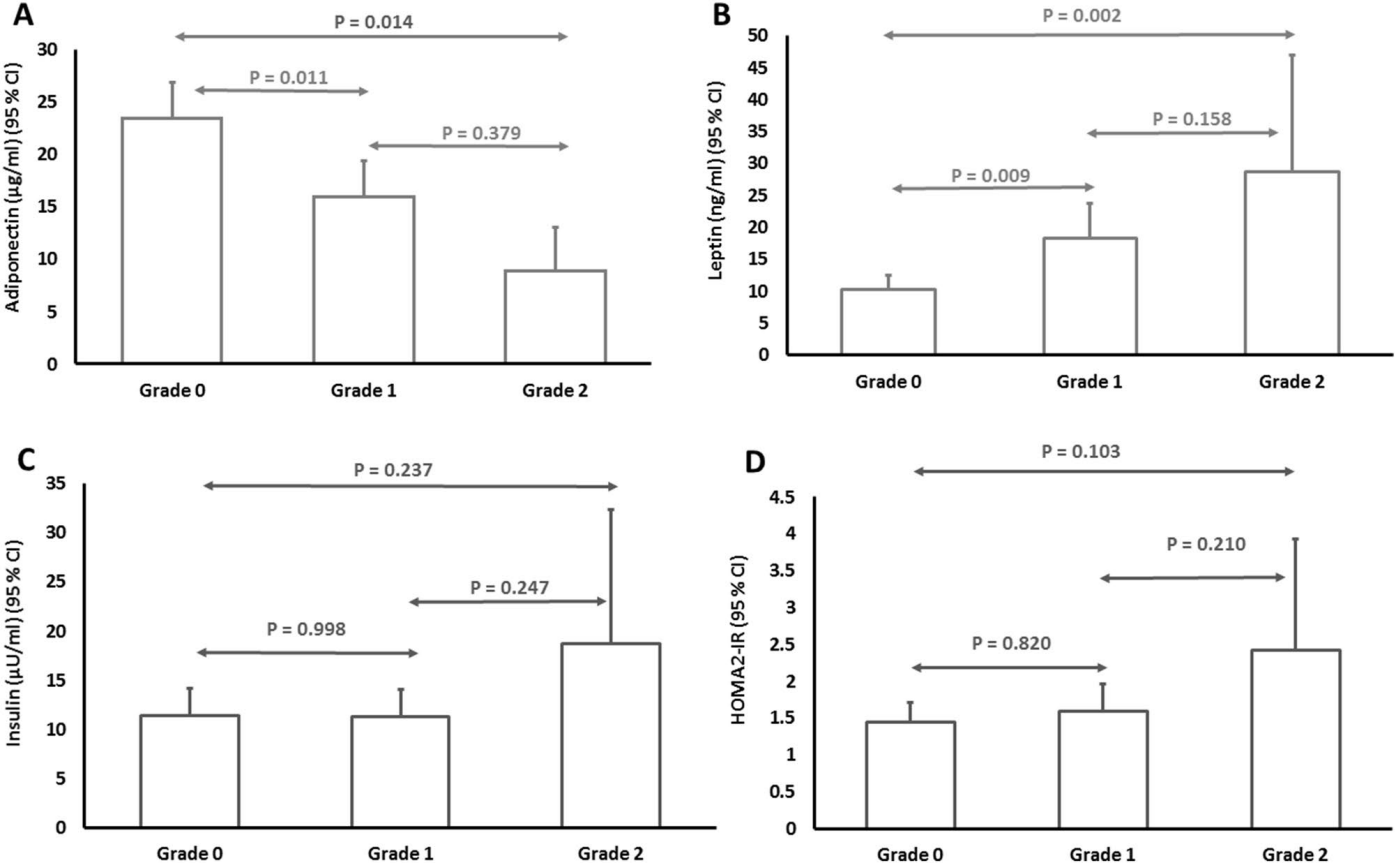
Comparison of serum adiponectin (**A**), serum leptin (**B**), serum insulin (**C**) and HOMA2-IR (**D**) based on different grades of steatosis in ultrasound.

Serum adiponectin level was statistically different over different grades of steatosis in ultrasound (F (2, 166) = 7.052, P = 0.001). Serum adiponectin level was significantly lower in patients with grade 2 steatosis (8.89 ± 6.52 µg/ml) and grade 1 steatosis (15.97 ± 12.72 µg/ml) compared to the patients without steatosis (23.48 ± 17.37 µg/ml). Serum leptin level was statistically different over different grades of steatosis in ultrasound (F (2, 166) = 8.698, P < 0.001). Serum leptin level was significantly higher in patients with grade 2 steatosis (28.74 ± 28.68 ng/ml) and grade 1 steatosis (18.37 ± 20.54 ng/ml) compared to the patients without steatosis (10.29 ± 11.53 ng/ml) (Fig. 2). Serum insulin level was not statistically different over different grades of steatosis (F (2, 166) = 1.390, P = 0.252) (Fig. 2).

### Liver fat contents measured by CAP are associated with serum adipokines and insulin resistance

**Figure 3:**
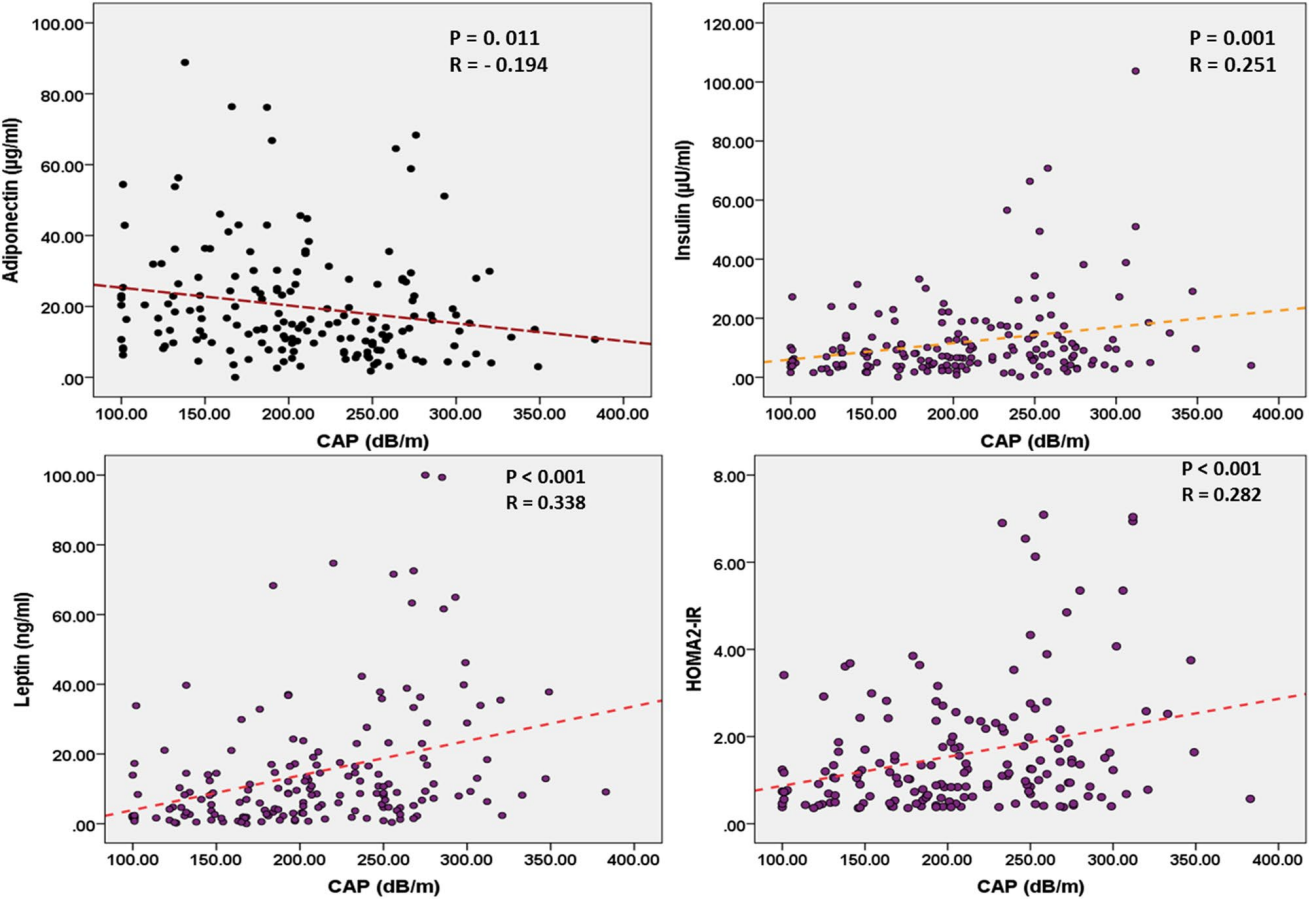
Correlation of controlled attenuation parameter with serum adiponectin, serum leptin, serum insulin and HOMA2-IR. Pearson’s test was used for calculation of correlation efficient (r) and P-value.

CAP was measured in 172 patients. Mean CAP values in our patients was 208.19 ± 61.64 dB/m. The correlation of CAP with serum adiponectin, insulin, leptin, HOMA2-IR and other metabolic indices are outlined in Table 3. Using Pearson correlation test, CAP was negatively correlated with serum adiponectin (P = 0.011) and positively correlated with serum insulin (P = 0.001), leptin (P < 0.001) and HOMA2-IR (P < 0.001) (Fig. 3). Serum tacrolimus level was not correlated with CAP values (r = − 0.102, P = 0.253). CAP was also positively associated with BMI (P < 0.001), serum triglyceride (P < 0.001) and FBS (P < 0.001) (Table 3). In linear regression analysis, serum adiponectin, leptin, HOMA-IR and serum triglyceride were independently associated with CAP (Table 3).

**Table 3:** Correlation of CAP with adiponectin, leptin, and insulin resistance.

CAP				
	Pearson correlation		Linear regression analysis	
	r	P-value	β	P-value
Adiponectin (µg/ml)	− 0.194	0.011	− 0.147	0.039
Leptin (ng/ml)	0.338	< 0.001	0.227	< 0.001
HOMA 2-IR	0.282	< 0.001	0.253	< 0.001
BMI (Kg/m^2^)	0.257	0.001	0.134	0.066
Cholesterol (mg/dL)	0.065	0.429		
Triglyceride (mg/dL)	0.364	< 0.001	0.293	< 0.001
HDL (mg/dL)	− 0.133	0.106		
LDL (mg/dL)	0.133	0.105		
Tacrolimus level	− 0.101	0.253		

*CAP* controlled attenuation parameter, *BMI* body mass index, *HOMA-IR* homeostatic model for insulin resistance, *HDL* high density lipoprotein, *LDL* low density lipoprotein.

**Figure 4:**
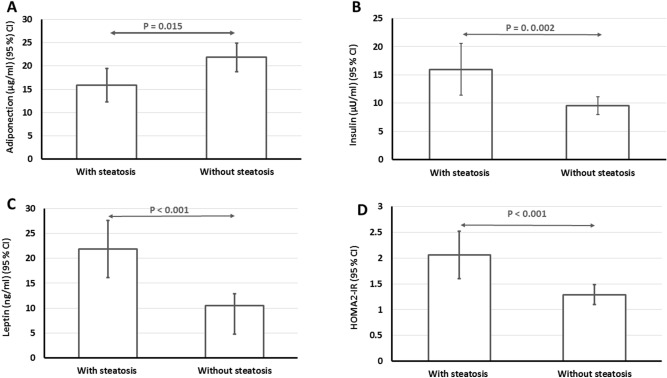
Comparison of serum adiponectin, leptin, insulin and HOMA2-IR in those with and without hepatic steatosis in transient elastography.

The association between serum adipokines and hepatic steatosis diagnosed by TE is outlined in Fig. 4. Patients with hepatic steatosis diagnosed by TE had significantly higher serum levels of leptin (P < 0.001), insulin (P = 0.002), HOMA2-IR (P < 0.001) and lower levels of serum adiponectin (P = 0.015) compared to the patients without hepatic steatosis in TE.

### Hepatic steatosis in lean patients after liver transplantation and the impact of low serum adiponectin

Ninety two patients (53.5%) were lean (BMI < 25 kg/m^2^). Among lean subjects, 20 patients (22.2%) had hepatic steatosis (11.2% of all the study population). In univariate analysis, post-transplant hyperlipidemia (odds ratio = 3.47: 95% CI 1.092–11.030; P-Value = 0.029), PTDM (odds ratio = 4.23: 95% CI 1.480–12.128; P-Value = 0.005), and low serum adiponectin level (P = 0.045) were associated with hepatic steatosis in lean liver transplant recipients (Table 4). In regression analysis, PTDM (odds ratio = 1.05: 95% CI 0.078–1.333; P-Value = 0.014), and low serum adiponectin level (odds ratio = 0.94: 95% CI 0.902–0.996; P-Value = 0.032) were independently associated with hepatic steatosis among lean liver transplant recipients (Table 4).

**Table 4:** Characteristics of lean patients with and without hepatic steatosis after liver transplantation.

	Univariate			Multivariate	analysis	
	With steatosis	Without steatosis	P-value	OR	95% CI	P-value
Age (year)	43.35 ± 12.66	41.26 ± 13.11	0.529			
Sex (male/female)	13/7	37/35	0.282			
WC(cm)	87.45 ± 6.86	86.47 ± 8.51	0.641			
HC (cm)	95.85 ± 6.21	95.73 ± 6.38	0.945			
BMI (kg/m^2^)	21.26 ± 2.46	21.60 ± 2.27	0.561			
TG (mg/dL)	151.17 ± 87.45	120.16 ± 68.35	0.126			
Cholesterol (mg/dL)	169.82 ± 63.56	163.43 ± 50.50	0.665			
AST (IU/L)	21.88 ± 8.52	33.91 ± 38.13	0.202			
ALT (IU/L)	27.17 ± 11.49	41.72 ± 57.24	0.303			
HTN, n (%)	4 (20)	11 (16.7)	0.731			
HLP, n (%)	7 (35)	9 (13.4)	0.029	0.30	0.081–1.111	0.072
PTDM, n (%)	11 (55)	15 (22.7)	0.005	1.05	0.078–1.333	0.014
Adiponectin (µg/ml)	15.10 ± 8.70	23.39 ± 17.56	0.045	0.94	0.902–0.996	0.032
Leptin (ng/ml)	9.70 ± 16.70	8.44 ± 12.50	0.715			
Insulin (µU/ml)	9.94 ± 9.03	8.90 ± 7.44	0.600			
HOMA2-IR	1.27 ± 0.99	1.23 ± 0.91	0.884			

*BMI* Body mass index, *WC* waist circumference, *HC* Hip circumference, *PTDM* Post transplant diabetes mellitus, *HLP* Hyperlipidemia, *HTN* Hypertension, *HOMA-IR* Homeostatic model for insulin resistance, *AST* Aspartate aminotransferase, *ALT* Alanine aminotransferase.

## Discussion

Our results demonstrate that hepatic steatosis diagnosed by ultrasound and TE in liver transplant recipients is associated with decreased serum adiponectin and increased serum leptin and insulin levels. Liver transplant recipients with hepatic steatosis had higher insulin resistance as assessed by HOMA2-IR. PTDM was also an independent predictor of hepatic steatosis after liver transplantation. We used CAP for non-invasive estimation of liver fat content. CAP values were different over different grades of hepatic steatosis in ultrasound and was correlated negatively with serum adiponectin and positively with serum leptin and HOMA2-IR. After adjustment for age, gender, BMI and other metabolic indices, serum adipokines and insulin resistance were independently associated with hepatic steatosis after liver transplantation highlighting their role in pathogenesis of hepatic steatosis after liver transplantation. In a subgroup of lean patients, decreased serum adiponectin level was an independent predictor of hepatic steatois after liver transplantation.

Recurrent or de novo hepatic steatosis after liver transplantation has been reported to be associated with PTDM, higher BMI and other components of metabolic syndrome^16–18^. However, limited data is available about pathogenesis of hepatic steatosis after liver transplantation. Increased fasting hepatic gluconeogenesis and impairment of suppression of hepatic glucose production are two components of hepatic insulin resistance^19^. Diacylglycerol (DAG) and ceramide have been suggested to be the two major lipids involved in hepatic insulin resistance via activation of protein kinase and downstream impairment of hepatic insulin signaling^20^. Total hepatic DAG and ceramide have been correlated with HOMA-IR as a marker of insulin resistance^21,22^. It is well known that insulin resistance is strongly associated with hepatic steatosis^23^. In a population based cohort, HOMA- IR was closely associated with liver fat contents assessed by magnetic resonance spectropscopy^24^. However, the role of insulin resistance in the development of hepatic steatosis after liver transplantation is controversial.

In contrast to our findings, Andrade and colleagues reported that HOMA-IR, as a marker of insulin resistance, was not a predictor of NAFLD after liver transplantation^25^. In this study, we used HOMA2-IR (the updated HOMA model) that is a better indicator of hepatic insulin resistance as it accounts for variations in both peripheral and hepatic glucose output^26^. Insulin resistance is prevalent among patients undergoing liver transplantation for hepatitis C virus (HCV) cirrhosis^27^ and other causes of liver cirrhosis^28^. A cross-sectional study showed that insulin resistance is highly prevalent and associated with new onset diabetes after liver transplantation^29^. None of these studies reported the impact of insulin resistance on the development of hepatic steatosis after liver transplantation.

Leptin and adiponectin are two major adipokines that are produced and secreted mainly from adipocytes and are involved in the pathogenesis of hepatic steatosis^15^. Leptin acts in liver cells via activation of the Janus kinase (JAK) 2/signal transducer and activator of transcription (STAT)-3 pathway resulting in increased expression of suppressor of cytokine signaling (SOCS)-3 which is a feedback inhibitor of leptin signaling^30^. It has been shown that inhibition of SOCS-3 improves leptin resistance and hepatic steatosis^31^. A meta-analysis of 33 studies has shown that serum leptin level was higher in patients with simple steatosis and NASH compared to the healthy controls^32^. Adiponectin is decreased when adipose tissue mass increases and has anti-steatotic properties on hepatocytes. Adiponectin promotes oxidation of free fatty acids, inhibits gluconeogenesis and prevents apoptosis of hepatocytes. It has also anti-fibrotic and anti-inflammatory effects by acting on Kupffer cells and hepatic satellite cells^33–35^.

Alterations of serum adipokines after liver transplantation have been reported previously in few studies, none of them focused on hepatic steatosis. Watt et al. reported that increased serum leptin and decreased serum adiponectin levels were seen in liver recipients with cardiovascular events^36^. A recent study reported that hypoadiponectinemia was an independent predictor of future cardiovascular events in liver transplant recipients^37^. However, a cross-sectional study among a small group of patients failed to demonstrate any correlation between serum adipokines and metabolic syndrome after liver transplantation^38^. In a previous study no association was demonstrated between hepatic steatosis and serum leptin after liver transplantation^39^. In the mentioned study, authors only included patients with HCV infection and did not evaluate the impact of serum adiponectin. It has been recently suggested that patients with NAFLD and adiponectin gene polymorphisms were susceptible to hepatic steatosis after liver transplantation^40^.

In this study, we showed, for the first time, that alterations in serum adiponectin and leptin as well as insulin resistance were correlated with hepatic steatosis and hepatic fat contents in liver transplant recipients independent of traditional risk factors of hepatic steatosis. We used ultrasound and CAP for non-invasive estimation of hepatic steatosis. CAP is a non-invasive, cheap and easy technique (compared to other methods) for quantitative measurement of liver fat content during transient elastography^41,42^. This parameter has been increasingly measured in patients with NAFLD^43^ and suggested to have good efficacy for assessment of hepatic steatosis after liver transplantation^44^.

Finally, cumulative evidence suggests that hepatic steatosis is prevalent after liver transplantation, however, its long term impact on allograft and patient outcomes is less evident. Our study expands current knowledge about pathophysiology of hepatic steatosis after liver transplantation. Liver transplant recipients with insulin resistance and alterations in adiponectin and leptin are susceptible to hepatic steatosis and should be actively diagnosed and treated. Targeting insulin resistance and adipokines can be considered as therapeutic options for prevention and management of hepatic steatosis after liver transplantation.

## Methods and materials

### Patients

In a cross-sectional study, all adult (> 18 years) liver transplant recipients who referred for their routine post-transplant follow-up between July 2017 and November 2017 were included in the study. All patients had undergone liver transplantation from deceased donors at Shiraz Organ Transplant Center, Shiraz, Iran. We included patients who have passed at least 6 months after transplant surgery. Tacrolimus based immunosuppressive regimen was used for the maintenance of immunosuppression after liver transplantation for all patients. Patients with acute rejection episodes and those with serum aminotransferase levels higher than 5 times upper limit of normal range were excluded from the study. Clinical characteristics of patients were recorded at the time of study inclusion. Body mass index (BMI) was calculated using this formula: weight (kg) divided by height (m^2^) squared. Patients with BMI < 25 kg/m^2^ were defined to be lean. Hepatic steatosis in transplant recipients was assessed by ultrasound and classified as grades 0, 1, 2, and 3 based on the severity of liver echogenicity as a marker of severity of hepatic steatosis.

### Measurement of serum adiponectin, leptin and insulin

Peripheral venous blood samples were collected from patients in the morning after an overnight fasting. Blood samples were centrifuged immediately at 4 °C and separated serum samples were stored at − 80 °C. Serum adiponectin levels were measured by enzyme linked immunoassay (ELISA) method using human adiponectin ELISA kit (Mediagnost, Reutlingen, Germany). Serum insulin levels were measured by ELISA method using human insulin kit (Monobind Inc., Lake Forest, CA (92630), USA). Serum leptin levels were measured using human leptin ELISA kit (Mediagnost, Reutlingen, Germany). Homeostatic model assessment of insulin resistance (HOMA-IR) is a method for assessment of insulin resistance and β-cell function based on the fasting plasma insulin and glucose concentrations. Insulin resistance was estimated using HOMA 2-IR software, as described before^45^. Blood sampling was performed on the day of clinical visit and assessment of hepatic steatosis by ultrasound and CAP measurement.

### Measurement of controlled attenuation parameter (CAP)

Non-invasive estimation of hepatic fat content was measured for all patients using vibration‐controlled transient elastography (VCTE) and expressed as CAP. Measurement of CAP was done on the day of clinical visit and blood sampling and after an overnight fasting. All CAP measurements was done by one person. CAP was obtained along the inter-axillary line and intercostal spaces on the liver while the patient was in supine position. CAP measurement was considered to be reliable if 10 valid successful acquisitions were made correctly. TE was performed using M probe in all patients and XL probe was used in obese patients in whom use of M probe was failed. CAP measurement was expressed in decibel per meter (dB/m)^46^. The cutoff scores for CAP estimation of hepatic steatosis were as follows: ≥ 238 dB/m for S1 (corresponding to 11–32% liver fat), ≥ 259 dB/m for S2 (33–65% liver fat), and ≥ 292 dB/m for S3 (≥ 66% liver fat), as previously determined.

### Statistical analysis

Continuous and categorical variables were compared using Student’s t-test and Chi-square test, respectively. Data were presented using means ± standard deviation for numeric variables, and percents and counts for categorical variables. A one way analysis of variance (ANOVA) and post-hoc Tukey test were used to compare serum levels of adiponectin, leptin and insulin with different grades of hepatic steatosis. Logistic regression analysis was used to identify the independent variables and association of serum adipokines and insulin resistance with hepatic steatosis after liver transplantation. Pearson’s and Spearman’s correlation coefficients were used to assess correlation of serum adipokines and insulin resistance with CAP values after liver transplantation. Linear regression analysis was used to identify continuous variables independently associated with CAP values. Statistical analysis was performed with SPSS 20.0 (SPSS Inc.; Chicago, IL, USA). A P- value of < 0.05 was considered statistically significant.

### Ethics and consent

The study protocol was approved by the institutional review board of Organ Transplant Center, Shiraz University of Medical Sciences on 5/11/2016. The study protocol was performed in accordance with the Helsinki Declaration as revised in Seoul 2008. Written informed consents were obtained from each patient after explaining the study harms and benefits.

## Data Availability

Due to patient privacy the datasets in the current study are not publicly available but can be available by corresponding author on reasonable request.

## Abbreviations

HOMA-IR: Homeostatic model assessment of insulin resistance
CAP: Controlled attenuation parameter
PTDM: Post-transplant diabetes mellitus
NAFLD: Non-alcoholic fatty liver disease
NASH: Non-alcoholic steatohepatitis
